# Endodontic Management of a Maxillary Premolar with a Rare Configuration (Three Buccolingually Positioned Canals in a Single Root) as Confirmed by Cone Beam Computed Tomography: a Case Report

**DOI:** 10.30476/DENTJODS.2021.90947.1538

**Published:** 2022-12

**Authors:** Ali Hamedi, Hamid Jafarzadeh, Sara Navabi

**Affiliations:** 1 Faculty of Dentistry, Zabol University of Medical Sciences, Zabol, Iran; 2 Faculty of Dentistry, University of Toronto, Toronto, Ontario, Canada and Dental Research Center, Mashhad University of Medical Sciences, Mashhad, Iran; 3 Dept. of Endodontics, Faculty of Dentistry, Mashhad University of Medical Sciences, Mashhad, Iran

**Keywords:** Anatomical variations, Cone beam computed tomography, Endodontic treatment, Maxillary first premolar, Root canal anatomy

## Abstract

Missed canals pose a potential risk in the treatment of teeth with anatomical variations, even when multi-angled radiographs have been used. Incomplete mechanical and chemical debridement of the root canal system can lead to treatment failure. Therefore, clinicians must have adequate knowledge of normal root canal systems as well as any possible variations in order to prevent any failure during or after the treatment of teeth with anatomical variations. Any case report of such rare variations would add to this critically required body of knowledge. The current case report presents the diagnosis and endodontic treatment of a maxillary first premolar with one root and three canals (one palatal and two buccal canals, all buccolingually positioned, bifurcating in the apical region), which was different from premolars with 3 canals reported up to now. This configuration describes an unusual root canal system for the maxillary first premolar and does not fit into any of the well-known root canal classification systems

## Introduction

The main purpose of root canal treatment is complete mechanical and chemical debridement of the root canal system so as to cleanse it from bacteria or infection, which is then followed by thorough obturation of the root canal system using biocompatible filling material [ 1 ]. Any complexity that jeopardizes this process could lead to endodontic treatment failure, which means that certain complicacies could pose a serious challenge for the clinician. Complexities such as abnormal root canal anatomy and presence of extra canals can cause treatment failure; therefore, clinicians must have adequate in-depth knowledge of normal root canal systems as well as any possible variation [ 2 - 5 ].

Despite the rarity of some complexities, clinicians must be informed of their possibility to be able to avoid endodontic failure [ 6 ]. Kartal *et al.* [ 7 ] reported that 8.66% of premolars had one, 89.64% had two, and 1.66% had three root canals. The presence of three canals in maxillary premolars is most commonly associated with the presence of three roots, a configuration that bears a resemblance to that of a miniature three-canalled maxillary molar. The existence of three canals in maxillary premolars with one root is a much less frequent occurrence, which is obviously hard to detect. The canal configuration of such single-rooted, three-canalled maxillary premolars often comprises mesiobuccal, distobuccal, and palatal canals [ 8 ]. Single-rooted, three-canalled maxillary premolars with two buccolingually positioned buccal canals and one palatal canal are rare, which makes it challenging to identify them using common radiographic methods or tactile sensation. The current case report presents the non-surgical endodontic treatment of a maxillary first premolar with a rare root canal system characterized by the presence of three canals in one root, all of which were positioned buccolingually.

## Case Presentation

**Figure 1:**
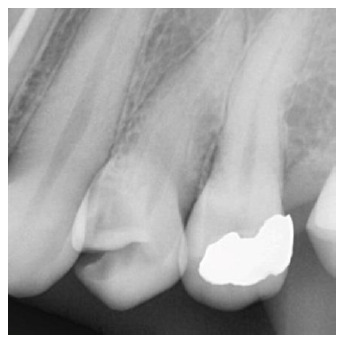
Pre-operative periapical radiograph of the left maxillary first premolar at the time of referral

A 32-year-old Iranian female with no history of medical problems was referred to the Department of Endodontics of Mashhad Faculty of Dentistry, Mashhad, Iran. The patient presented with a highly decayed left maxillary first premolar. Her chief complaint concerning this tooth was sensitivity to cold. Upon clinical examination, the tooth proved not sensitive to percussion and palpation, but showed normal mobility. Sensitivity to cold was confirmed clinically (FriscoSpray, Adarztbedarf GmbH, Frechen, Germany), and the tooth demonstrated a positive response to the electric pulp testing (Parkell Inc., Edgewood, New York). The periapical region was normal, as presented in the radiograph taken pre-operatively (Figure 1). 

**Figure 2:**
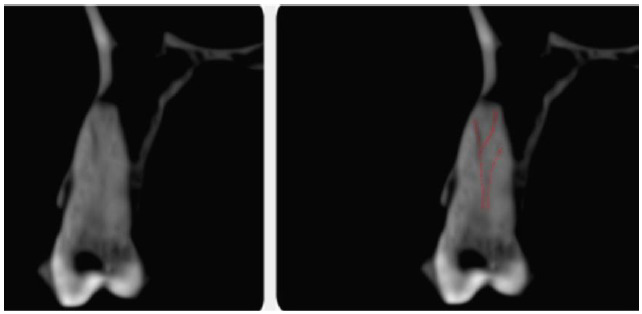
Cone beam computed tomography (CBCT) image of the tooth (coronal view)

**Figure 3:**
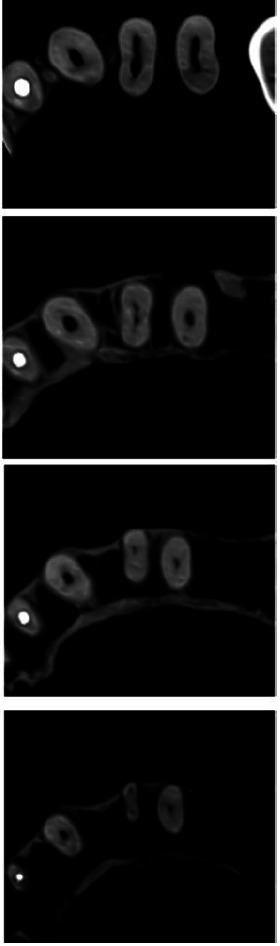
Cone beam computed tomography (CBCT) image of the tooth (axial view)

Based on the clinical and radiographic data, a diagnosis of chronic irreversible pulpitis was made. Accordingly, a non-surgical root canal treatment was planned for the tooth to be performed over two visits. Informed consent was obtained from the patient prior to intervention. In the first appointment, local anesthesia was administered with 1.8 mL of 2% lidocaine containing 1:100,000 epinephrine (Daroupaksh, Tehran, Iran) using the infiltration technique. After isolation with rubber dam, the infected enamel and dentin were removed. The access cavity was prepared using a diamond bur (Hager and Meisinger Inc., Neuss, Germany). The root canal system was then inspected using #10 K-files (Mani, Tochigi, Japan). Upon exploring, the root canal showed a peculiar anatomy, and so, the presence of additional canals was suspected. Thus, after the initial pulpectomy, a calcium hydroxide paste (Meta Biomed Co. Seoul, Korea) was placed as an intracanal medicament, and the tooth was temporarily restored with Cavit (3M ESPE, St. Paul, Mn, USA). Next, the patient was sent to the Department of Oral and Maxillofacial Radiology to take a relevant cone beam computed tomography (CBCT) image (ProMax 3D MAX; Planmeca OY, Helsinki, Finland). As evident in the CBCT view, despite the presence of only one orifice (one root), three canals were found in the observed area; one on the palatal side, and two on the buccal side, which appeared to be positioned buccolingually. The buccal canal itself divides into two canals in the apical third, as can be seen in the coronal view of the CBCT Image (Figure 2). The root canal transition and bifurcation were evident in multiple sections (from coronal to apical) in the axial view of the CBCT image (Figure 3).

**Figure 4:**
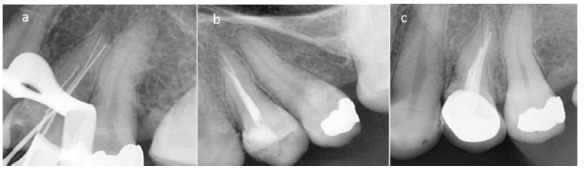
Periapical radiograph of the tooth with the restoration at the 12-month follow-up

In the second appointment, local anesthesia was administered with 1.8 mL of 2% lidocaine containing 1:1-00,000 epinephrine (Daroupaksh, Tehran, Iran) utilizing the infiltration technique. Isolation was accomplished with a rubber dam, and the temporary restoration was removed. The working lengths of the three root canals were determined using an apex locator (RayPex 6; VDW, Munich, Germany) and were confirmed with a radiograph. A #10 Hedstrom file (Mani, Tochigi, Japan) was initially applied in the palatal canal in order to 

make the distinction amongst the three canals easier (Figure 4a). 

The canals were mechanically prepared using the ProTaper Gold system (Dentsply Tulsa Dental, Tulsa, OK, USA) under copious irrigation with 5.25% sodium hypochlorite. The canals were properly cleaned and then shaped to a ProTaper F2. Patency was achieved and maintained in each canal with#10 K-file (Dentsply, Maillefer, Switzerland). The smear layer was removed using 17% ethylenediaminetetraacetic acid (EDTA) (Cerkamed, Stalowa Wola, Poland) for one minute.

Sterile paper points (AriaDent, Tehran, Iran) were applied to dry the canals thoroughly. After that, root canal obturation was performed using the cold lateral compaction method with AH26 sealer (Dentsply, Maillefer, Switzerland) and gutta-percha (Meta Biomed Co., Seoul, Korea). The tooth was temporarily restored with Cavit (3M ESPE, St. Paul, MN, USA) and the patient was referred to a prosthodontist (Figure 4b).

The tooth was later restored with a porcelain-fused-to-metal (PFM) crown. A 12-month follow-up radiograph revealed no abnormalities, confirming a successful treatment (Figure 4c).

## Discussion

This case report presented a maxillary first premolar with one root and three canals; one palatal and two buccal canals, which were positioned buccolingually. This unusual root canal system does not fit into any of the well-known root canal classification systems. It has been reported that three-canalled maxillary premolars (with a prevalence rate of 6%) have one palatal canal and two mesiodistally-positioned buccal canals [ 8 ], which is a configuration different from that of the present case. Although such anatomy can be difficult to diagnose, the treatment itself does not constitute much of a challenge due to ease of the negotiation and debridement procedures. The unusual variation found in the present case can cause difficulties in the treatment process, which might result in iatrogenic failures if the clinician is not aware of the possibility of such a root canal system. The treatment of the present case faced complexities during canal negotiation and preparation. Inspite of the excellent guidance provided by CBCT, the clinical challenges of this peculiar root canal anatomy persisted.

Any variation in the root canal system is an important factor that can influence the steps involved in the endodontic treatment process (diagnosis, treatment planning, and prognosis). Additional information about the root canal system could be gathered using: magnifying devices, radiographs, a proper access cavity, and a thorough investigation of the root canal system. However, in cases with variations, there is always the possibility of missing a canal, even with the help of radiographs from different angles. CBCT imaging makes the detection of such elusive canals possible [ 9 ]. The unusual root canal system of the present case was primarily suspected through tactile sensation. However, it could not have been confirmed using multi-angled periapical radiographs, and therefore, CBCT imaging was indicated for confirmation prior to root canal preparation. The coronal view of the tooth confirmed the suspected root canal system. Hence, it can be suggested that the coronal view of the CBCT scan could provide help in cases where a canal system similar to that of the current case is anticipated.

The application of CBCT as a diagnostic modality can bring many advantages to endodontics. It can be used, for example, to ensure successful evaluation of the root canal system and its possible variations. It can also help to identify root fractures, establish the presence of pathosis in the area, and detect resorptive lesions. It has a short scan time and markedly reduces the radiation dose [ 10 - 13 ]. CBCT scans tend to reveal a higher number of unidentified root canals when compared with conventional radiography [ 14 ]. Therefore, CBCT imaging appears to be of importance, especially when a complex root canal system is suspected [ 15 ]. 

The root canal treatment of maxillary first premolars could present a challenge even for the most experienced endodontists due to the variations in their anatomy, including: the number of roots; the number of canals; the differences in canal and pulp chamber configurations; the various longitudinal and directional depressions on the roots; and the difficulty in visualization of the apical anatomy on radiographs [ 16 - 19 ]. A maxillary first premolar is the only tooth that can exhibit all eight types of Vertucci’s classification (20). In a study by Ashegi *et al.* [ 21 ] conducted on the root canal variations of maxillary first premolars in an Iranian population, 1.73% of the teeth had three roots, 48% had two roots, and 50% were single-rooted. Although maxillary first premolars commonly have two canals, it has been shown by several studies that three canals can exist in up to 6% of these teeth [ 16 , 19 , 22 ].

Despite the comprehensive efforts to classify different root canal systems, rare and uncharted canal configurations, as observed in the present case, are still possible to be discovered in everyday endodontic practice. This highlights the need for clinicians to be aware of different root canal morphologies as a prerequisite for optimum debridement and disinfection of the root canal systems, thereby ensuring a satisfying outcome [ 23 ]. 

An informed consent was taken from the patient regarding the publishing of digital intraoral radiographs, CBCT and medical history.

## Conclusion

This case report presented the endodontic management of a maxillary first premolar with one root and three root canals; one palatal canal and two buccal canals that were positioned buccolingually. Further similar case reports will certainly add to the much-needed body of knowledge about maxillary first premolars and their variations, which could in turn contribute to a better understanding of the complexities of their root canal systems, promising a higher success rate of the ensuing treatment.

## Conflict of Interest

The authors declare that they have no conflict of interest.
